# Are computed tomography-based measures of specific abdominal muscle groups predictive of adverse outcomes in older cancer patients?

**DOI:** 10.1016/j.heliyon.2020.e05437

**Published:** 2020-11-09

**Authors:** S.M.L.M. Looijaard, A.B. Maier, A.F. Voskuilen, T. Van Zanten, D.E. Bouman, J.M. Klaase, C.G.M. Meskers

**Affiliations:** a Department of Human Movement Sciences, @AgeAmsterdam, Faculty of Behavioural and Movement Sciences, VU University, Amsterdam Movement Sciences, Van der Boechorststraat 7, 1081 BT, Amsterdam, the Netherlands; b Department of Medicine and Aged Care, @AgeMelbourne, The Royal Melbourne Hospital, University of Melbourne, 300 Grattan Street, Parkville, Melbourne, 3050, Victoria, Australia; c Department of Radiology, Medical Spectrum Twente, Koningstraat 1, 7512 KZ, Enschede, the Netherlands; d Department of Surgery, Medical Spectrum Twente, Koningstraat 1, 7512 KZ, Enschede, the Netherlands; e Department of Hepatobiliary Surgery and Liver Transplantation, University Medical Center Groningen, Hanzeplein 1, 9713 GZ, Groningen, the Netherlands; f Department of Rehabilitation Medicine, Amsterdam University Medical Center, VU University Medical Center, Amsterdam Movement Sciences, De Boelelaan 1118, 1081 HZ, Amsterdam, the Netherlands

**Keywords:** Medical imaging, Abdominal surgery, Cancer surgery, Digestive system, Gastrointestinal system, Internal medicine, Musculoskeletal system, Oncology, Body composition, Computed tomography, Sarcopenia, Surgery, Aged, Neoplasms

## Abstract

**Purpose:**

It is unknown whether computed tomography (CT)-based total abdominal muscle measures are representative of specific abdominal muscle groups and whether analysis of specific abdominal muscle groups are predictive of the risk of adverse outcomes in older cancer patients.

**Methods:**

Retrospective single-center cohort study in elective colon cancer patients aged ≥65 years. CT-based skeletal muscle (SM) surface area, muscle density and intermuscular adipose tissue (IMAT) surface area were determined for rectus abdominis; external- and internal oblique and transversus abdominis (lateral muscles); psoas; and erector spinae and quadratus lumborum (back muscles). Outcomes were defined as severe postoperative complications (Clavien-Dindo score >2) and long-term survival (median follow-up 5.2 years).

**Results:**

254 older colon cancer patients were included (median 73.6 years, 62.2% males). Rectus abdominis showed the lowest SM surface area and muscle density and the back muscles showed the highest IMAT surface area. Psoas muscle density, and lateral muscle density and percentage IMAT were associated with severe postoperative complications independent of gender, age and cancer stage.

**Conclusions:**

CT-based total abdominal muscle quantity and quality do not represent the heterogeneity that exists between specific muscle groups. The potential added value of analysis of specific muscle groups in predicting adverse outcomes in older (colon) cancer patients should be further addressed in prospective studies.

## 1 Introduction

Routine single-slice abdominal computed tomography (CT)-based muscle measures at the abdominal, third lumbar vertebra level such as total cross-sectional skeletal muscle (SM) surface area, muscle density and intermuscular adipose tissue (IMAT) surface area, are increasingly being used to predict adverse outcomes in (geriatric) oncology. However, studies on the association of CT-based muscle measures and adverse outcomes including postoperative complications, chemotherapy toxicity and survival in older colon cancer patients, show inconsistent results [1, 2, 3, 4]. It is unknown whether total abdominal muscle measures can be assumed representative of specific abdominal muscle groups and whether analysis of specific muscle groups is of clinical value in predicting the risk of adverse outcomes in older cancer patients.

There are morphological differences in the effects of age on specific muscle groups [5, 6, 7]. Muscle surface area and muscle density of superficial muscles such as the rectus abdominis and external- and internal oblique muscles are affected more with age in terms of atrophy and muscle density than deep muscles and back muscles such as the transversus abdominis and erector spinae muscles [6, 8, 9]. On the other hand, deep muscles and back muscles appear to be more affected by inactivity, as is shown in older women during twelve months of institutionalization, and in whom the erector spinae muscle was mainly affected by atrophy [9, 10]. The value of analyzing specific muscle groups through CT scan analysis has not been studied in the field of (geriatric) oncology.

In a cohort of older colon cancer patients we addressed: 1) whether CT-based muscle measures SM surface area, muscle density and IMAT surface area significantly differed between specific abdominal muscle groups; 2) the intra-individual heterogeneity for each muscle measure and its determinants; and 3) the association of measures of specific muscle groups and intra-individual heterogeneity with the risk of severe postoperative complications and long-term survival.

## 2 Materials and methods

### 2.1 Study population

This study included colon cancer patients of the retrospective single-center cohort study PREdictive value of MUScle mass in CoLorectal cancer in Elderly (PREMUSCLE). The PREMUSCLE study encompassed 378 primary colorectal cancer patients of ≥65 years old who received elective surgery between 2010-2014 at Medical Spectrum Twente, a large teaching hospital in the Netherlands. The PREMUSCLE study was specifically aimed at older cancer patients as older patients are known to have a higher risk of adverse outcomes [11, 12] and are more often affected by poorer skeletal muscle status [13]. Patients were selected from the Dutch ColoRectal Audit (DCRA), which contains prospectively collected information on patient- and tumor characteristics, treatment and outcomes of colorectal surgery (DICA, 2017). Patient data was retrieved from the DCRA and hospital information system and included gender, age, body weight, height, preoperative number of comorbidities and medications (medical record review), Karnofsky Performance Scale score, tumor- and treatment-related characteristics and pre-defined outcome measures. Survival data was extracted from the civil registry. Exclusion criteria for the PREMUSCLE study were ≥2 primary tumors requiring surgery, benign or non-colorectal tumor, surgery classified as acute, urgent or elective after stent placement, missing data on body height, weight or outcomes, or no eligible preoperative CT scan at the third lumbar vertebra (L3) available. This excluded patients of whom no CT scan was available, CT scans showed quality defects and if total SM surface area could not be determined. For this study, only patients with colon cancer were included (N = 284) to increase the homogeneity of the study population as type of cancer may affect type of treatment and related type of complications, muscle parameters and risk of adverse outcomes. Rectal cancer patients (N = 94) and patients with CT scans not suitable for specific muscle group analysis (N = 30) were excluded. Patients with high-risk stage 2 and stage 3 colon cancer were considered for adjuvant chemotherapy. These patients were discussed by a multidisciplinary team to decide whether a patient was referred to the Medical Oncology department to discuss possible adjuvant chemotherapy treatment. Therefore, part of the included patients in this study may have received adjuvant chemotherapy after elective surgery. More information on the PREMUSCLE study has been published elsewhere [14]. The PREMUSCLE study was approved by the Medical Ethics Committee of the Amsterdam University Medical Center, location VU Medical Center, with site-specific approval of Medical Spectrum Twente. Informed Consent was waived due to the retrospective design of the study.

### 2.2 CT scan analysis

CT scans were obtained preoperatively for staging purposes and analyzed using medical imaging software SliceOMatic version 5.0 (TomoVision, Montreal, QC, Canada), which identifies body tissues based on Hounsfield units (HU). CT scan selection and analyses were performed by a trained and certified researcher (SL) for the PREMUSCLE study, as previously described [14]. Muscle measures were determined by analyzing single-slice abdominal CT scans of L3 [15,16]. Tube potential varied between 100-120 kV and slice thickness between 1-5 mm. Contrast-enhanced CT scans were used. The median number of days between CT scan and surgery was 31 [interquartile range (IQR) 23.0–42.0 days].

**Figure 1:**
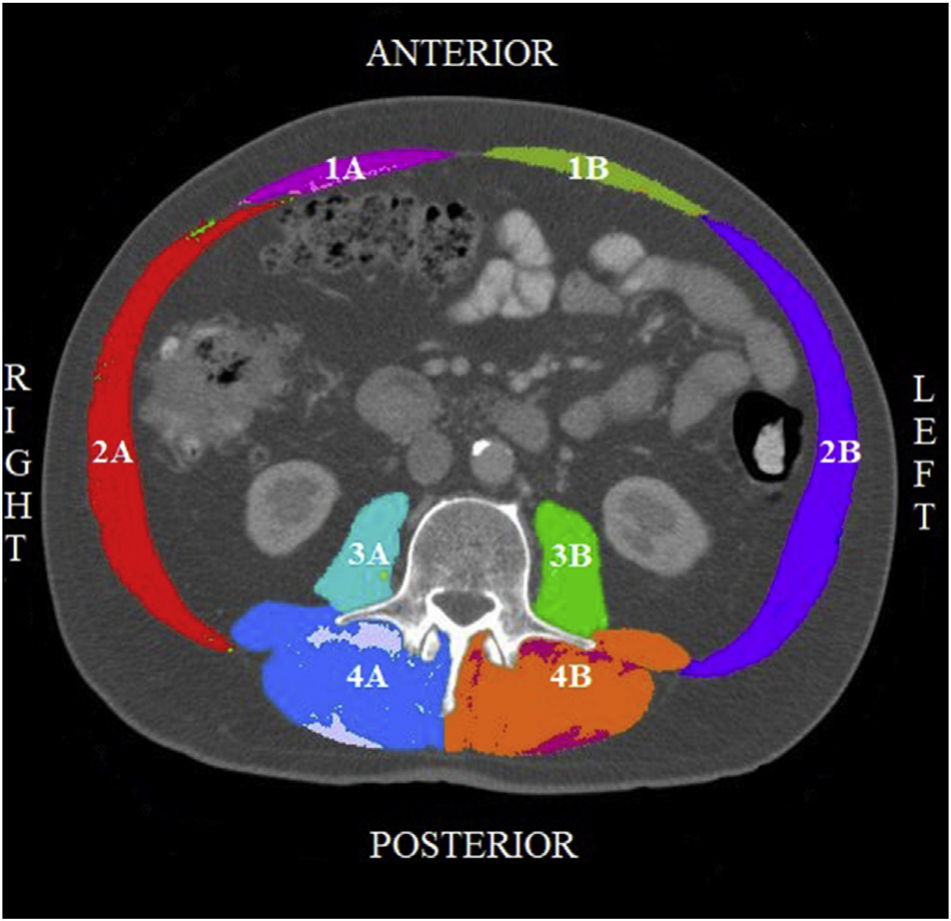
Coding of specific muscle groups. Computed tomography scan of the level of the third lumbar vertebra. Skeletal muscle and intermuscular adipose tissue of specific muscle groups have been identified in different (color) coding. Muscle groups from anterior to posterior: 1) rectus abdominis muscle; 2) lateral muscles: external- and internal oblique muscles and transversus abdominis muscle; 3) psoas muscle; and 4) back muscles: erector spinae muscle and quadratus lumborum muscle. A: right side; B: left side.

Two trained users of SliceOMatic (AV and TZ) determined the boundaries of specific muscles and analyzed SM and IMAT of each muscle by color coding. HU ranges for SM were set between -29 and +150 HU and between -190 and -30 HU for IMAT [15, 17]. Four specific muscle groups were discerned: 1) rectus abdominis, 2) lateral muscles: external- and internal oblique and transversus abdominis, 3) psoas, and 4) back muscles: erector spinae and quadratus lumborum muscles. External- and internal oblique and transversus abdominis were analyzed as one muscle, as well as the erector spinae and quadratus lumborum muscles, since these muscles could hardly be distinguished on CT scans. Figure 1 shows an example of a CT scan in which the color coding is illustrated. CT scans were excluded if 1) muscle measures could not be determined bilaterally due to quality defects or frame selection (N = 27); and 2) unilateral boundaries of more than two muscle groups could not be distinguished from each other (N = 3). In case boundaries of one or two muscle groups could not be determined unilaterally, contralateral surface area was multiplied. Boundaries of muscle groups were double-checked for every CT scan (AV and TZ) and in case of doubt a third assessor was consulted (SL). The inter-observer correlation coefficient for variability of SM, muscle density and IMAT was between 0.98-1.00 using a single measure two-way mixed model with absolute agreement based on a random selection of 10% (N = 26).

The total cross-sectional surface area in cm^2^ was computed by multiplying pixel area with the number of pixels. Percentage of SM surface area was calculated by dividing SM surface area of a specific muscle group by total SM surface area multiplied by 100; percentage IMAT surface area by dividing IMAT surface area by the sum of SM and IMAT multiplied by 100. Muscle density, a measure of muscle quality determined by fat infiltration [18] was determined by the mean HU of muscle groups.

### 2.3 Clinical outcome measures

Severe postoperative complications were defined as any grade 3 (requiring re-intervention), grade 4 (requiring intensive care unit admittance) or grade 5 (death) surgical or medical complications according to the Clavien-Dindo Classification of Surgical Complications, during admission or within 30 days after surgery [19]. Long-term survival was calculated from day of surgery until day of death and was classified as died (all-cause mortality) or censored (alive on the 23^rd^ of April 2019). The median follow-up was 5.2 [IQR 3.8–6.5] years.

### 2.4 Statistical analyses

Variables were described by number (percentage), mean ± standard deviation (SD) or median [IQR]. Right-left symmetry was assessed by Pearson's correlation coefficient for normally distributed data (SM surface area and muscle density) and Spearman's correlation coefficient for skewed data (IMAT surface area). If aforementioned correlation coefficients were >0.3 indicating at least medium correlation [20] right and left side of muscles were grouped in further analyses. Differences in percentage of SM surface area and muscle density between specific muscle groups were tested by one-way repeated measures analysis of variance (ANOVA) and for percentage IMAT surface area by Friedman test. Assumption of sphericity was tested using Mauchly's test of sphericity and in case of violation, a Greenhouse-Geisser correction was applied. Any outliers defined as 1.5 IQRs from the 75^th^ percentile were checked for their genuineness and if plausible kept in the analyses. Post hoc tests used Bonferroni adjustment. Intra-individual heterogeneity of muscle measures across specific muscle groups was determined by calculating the SD per muscle measure per individual. This was calculated by: √(∑ ((value specific muscle rectus abdominis/lateral muscles/psoas/back muscles – mean of all four muscles)^2^)/(N-1)). Though percentage of IMAT surface area was not normally-distributed, non-log transformed values were used to calculate the SD to permit comparison with SM surface area and muscle density. Determinants of intra-individual heterogeneity were analyzed using linear regression analysis. Associations between specific muscle group measures and intra-individual heterogeneity, and severe postoperative complications and long-term survival were analyzed using logistic regression and cox proportional hazards model, respectively. All analyses were performed in a crude model and an adjusted model for age (continuous variable), gender (dichotomous variable) and stage of cancer (categorical variable) as these characteristics could affect both body composition and risk of adverse outcomes. P-values of <0.05 were considered statistically significant. Statistical analyses were performed using IBM SPSS Statistics version 25 (IBM SPSS Statistics, Feltham, UK).

## 3 Results

### 3.1 Patient characteristics

The median age of the 254 included patients was 73.6 years [IQR 69.7–78.5 years] and 158 patients were male (62.2%). Two or more comorbidities were present in 158 patients (62.2%). A total of 118 patients had high risk stage 2 or stage 3 colon cancer and were therefore considered for adjuvant chemotherapy treatment. Of these patients, 49 actually received adjuvant chemotherapy treatment (41.5%). The majority of patients underwent laparoscopic surgery (66.5%). Severe postoperative complications occurred in 29 patients (11.4%) and after a median of 5.2 years, 90 patients (35.4%) had died. Patient characteristics are shown in Table 1.

**Table 1:** Patient characteristics.

Patient characteristics	All patients
*Demographics*	
Gender, male	158 (62.2)
Age, years, median [IQR]	73.6 [69.7–78.5]
Number of comorbidities, ≥ 2	158 (62.2)
Number of medications, ≥ 5^a^	106 (42.1)
Karnofsky score, median [IQR]^b^	90.0 [80.0–100.0]
Cancer stage	
Stage 1	52 (20.5)
Stage 2	86 (33.9)
Of which high risk stage 2	35 (40.7)
Stage 3	83 (32.7)
Stage 4	23 (9.1)
Stage T0/unknown	10 (3.9)
Surgical approach, laparoscopic	169 (66.5)
Adjuvant chemotherapy^c^, yes	49 (41.5)
*Body composition*	
Height, cm, mean ± SD	170.3 ± 8.8
Body weight, kg, mean ± SD	79.7 ± 14.0
BMI, kg/m^2^, mean ± SD	27.4 ± 4.0
*Outcome measures*	
Postoperative complication, yes	108 (42.5)
Severe postoperative complications	29 (11.4)
Long-term survival, died	90 (35.4)

IQR: interquartile range; cm: centimeters; SD: standard deviation; kg: kilograms; BMI: body mass index; m: meters. All variables are described as number (percentage) unless indicated otherwise. Severe postoperative complications were defined as grade 3–5 complications according to Clavien-Dindo. Data available in N = 254 and a subgroup of ^a^ N = 252; ^b^ N = 238. ^c^Only patients with high risk stage 2 and stage 3 cancer were considered eligible for adjuvant chemotherapy treatment (N = 118).

### 3.2 Measures of specific muscle groups

Since all correlation coefficients between the right and left side of muscle groups showed at least a medium correlation, both sides were grouped in further analyses (Table 2). Table 3 shows the measures of specific muscle groups. Mean SM surface area varied between 9.7 cm^2^ and 51.5 cm^2^ and was the lowest for the rectus abdominis muscle and the highest for the back muscles. Mean muscle density varied between 18.3 HU for the rectus abdominis muscle and 45.1 HU for the psoas muscle. Median IMAT surface area and percentage respectively varied between 0.1 cm^2^ (0.6%) for the psoas muscle and 9.2 cm^2^ (15.6%) for the back muscles.

**Table 2:** Correlation between right and left side of specific muscle groups.

Muscle measures	N	All patients	Correlation coefficient	p-value
*Rectus abdominis muscle*				
SM, cm^2^, right side	251	4.9 ± 2.4		
SM, cm^2^, left side	250	4.8 ± 1.9	.61	**<0.001**
Muscle density, HU, right side	251	18.0 ± 14.7		
Muscle density, HU, left side	250	18.3 ± 13.8	.74	**<0.001**
IMAT, cm^2^, right side	251	0.2 [0.0–0.8]		
IMAT, cm^2^, left side	250	0.2 [0.0–0.8]	.54	**<0.001**
*Lateral muscles*				
SM, cm^2^, right side	251	25.1 ± 6.8		
SM, cm^2^, left side	250	24.5 ± 6.2	.89	**<0.001**
Muscle density, HU, right side	251	25.0 ± 9.7		
Muscle density, HU, left side	250	25.5 ± 9.4	.91	**<0.001**
IMAT, cm^2^, right side	251	1.4 [0.5–3.8]		
IMAT, cm^2^, left side	250	1.3 [0.5–2.9]	.84	**<0.001**
*Psoas muscle*				
SM, cm^2^, right side	254	8.2 ± 2.7		
SM, cm^2^, left side	253	8.7 ± 2.7	.92	**<0.001**
Muscle density, HU, right side	254	45.3 ± 9.4		
Muscle density, HU, left side	253	44.7 ± 9.2	.89	**<0.001**
IMAT, cm^2^, right side	254	0.0 [0.0–0.1]		
IMAT, cm^2^, left side	253	0.0 [0.0–0.1]	.47	**<0.001**
*Back muscles*				
SM, cm^2^, right side	254	25.4 ± 6.0		
SM, cm^2^, left side	254	26.1 ± 6.2	.92	**<0.001**
Muscle density, HU, right side	254	32.6 ± 9.4		
Muscle density, HU, left side	254	32.9 ± 9.1	.89	**<0.001**
IMAT, cm^2^, right side	254	4.6 [3.2–6.6]		
IMAT, cm^2^, left side	254	4.3 [3.1–6.3]	.79	**<0.001**

SM: skeletal muscle; cm: centimeters; HU: Hounsfield units; IMAT: intermuscular adipose tissue. The correlation coefficient was calculated with Pearson's correlation coefficient for SM surface area and muscle density and Spearman's rho for IMAT surface area. SM and muscle density are given as mean ± standard deviation and IMAT as median [interquartile range]. P-values of <0.05 were considered statistically significant and are indicated in bold.

**Table 3:** Measures of specific muscle groups.

Muscle measures	Rectus abdominis muscle	Lateral muscles	Psoas muscle	Back muscles
SM, cm^2^	9.7 ± 3.9	49.6 ± 12.7	16.9 ± 5.3	51.5 ± 11.9
SM, %	7.5 ± 2.2	38.8 ± 3.8	13.2 ± 2.6	40.5 ± 4.2
Muscle density, HU	18.3 ± 13.4	25.4 ± 9.4	45.0 ± 9.1	32.7 ± 9.0
IMAT, cm^2^	0.6 [0.1–1.6]	2.7 [1.0–6.8]	0.1 [0.0–0.2]	9.2 [6.4–12.9]
IMAT, %	5.7 [1.2–16.2]	5.4 [1.9–12.5]	0.6 [0.2–1.3]	15.6 [11.0–21.1]

SM: skeletal muscle; cm: centimeters; HU: Hounsfield units; IMAT: intermuscular adipose tissue. SM, SM % and muscle density are given as mean ± standard deviation and IMAT and IMAT % as median [interquartile range]. The right and left side of muscle groups were grouped in analyses.

There was an overall statistically significant difference between muscle groups for percentage of SM surface area, *F* (2.125, 537.660) = 5127.571, p < 0.001 and muscle density, *F* (2.097, 530.662) = 693.855, p < 0.001. All muscle groups differed from one another in percentage of SM surface area and muscle density (p < 0.001). Percentage IMAT surface area showed statistically significant differences between muscle groups (χ^2^ [3] = 435.907, p < 0.001), for all muscles (p < 0.001) except for the rectus abdominis muscle and lateral muscles (p > 0.99).

### 3.3 Intra-individual heterogeneity

**Figure 2:**
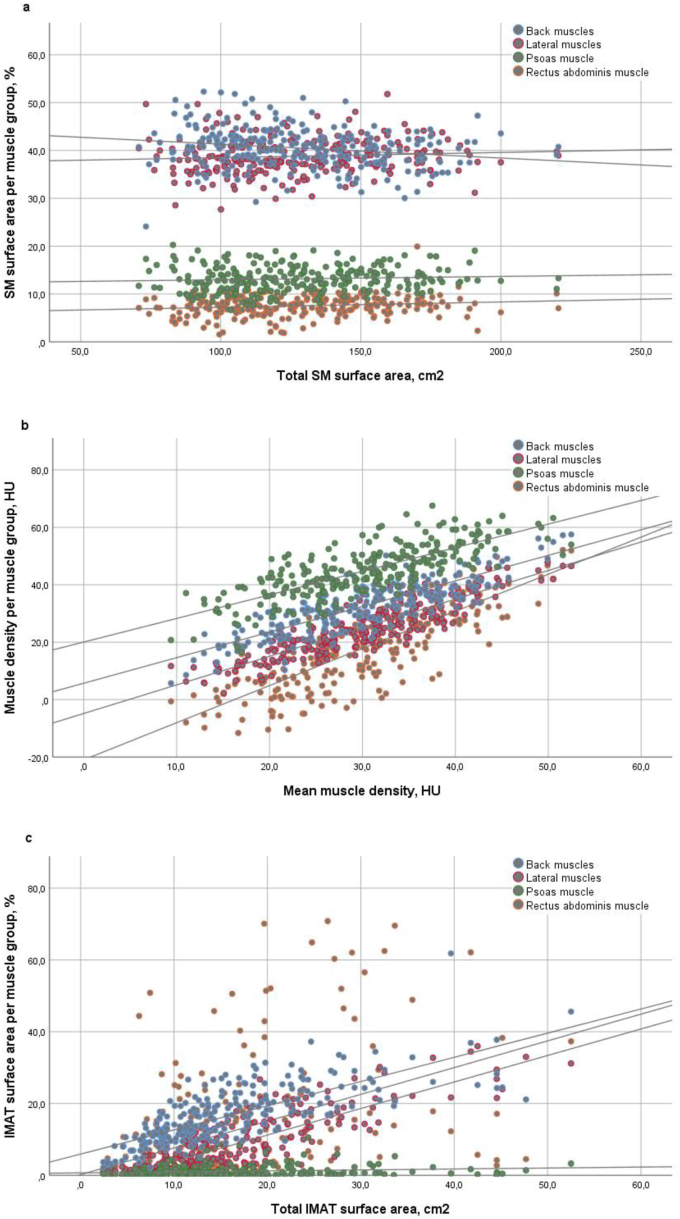
Intra-individual heterogeneity of muscle measures across specific muscle groups. SM: skeletal muscle; cm: centimeters; HU: Hounsfield units; IMAT: intermuscular adipose tissue. Intra-individual heterogeneity is shown for a) percentage of SM surface area per muscle group and total SM surface area; b) muscle density per muscle group and mean muscle density; and c) percentage IMAT surface area per muscle group and total IMAT surface area.

Intra-individual heterogeneity of percentage of SM surface area ranged from 12.5 to 22.9 SD (mean 17.4 SD), of muscle density from 1.2 to 25.6 SD (mean 12.4 SD) and from 0.8 to 30.3 SD (mean 9.2 SD) of percentage IMAT surface area. Intra-individual heterogeneity of SM surface area and muscle density was lower with higher total SM surface area and mean muscle density, respectively. Intra-individual heterogeneity of percentage IMAT surface area was higher with higher total IMAT surface area (Figure 2a-c). Higher age was associated with higher intra-individual heterogeneity in percentage of SM surface area (*β* = 0.061, p = 0.002) and percentage IMAT surface area (*β* = 0.216, p < 0.001). Higher Karnofsky score was associated with a lower intra-individual heterogeneity in percentage of SM surface area (*β* = -0.031, p = 0.008), muscle density (*β* = -0.066, p = 0.03) and percentage IMAT surface area (*β* = -0.075, p = 0.04) (Table 4).

**Table 4:** Determinants of intra-individual heterogeneity of muscle measures across specific muscle groups.

Determinants	SM %, SD			Muscle density, SD			IMAT %, SD		
	*β*	SE	p-value	*β*	SE	p-value	*β*	SE	p-value
Age, years	**0.061**	**0.019**	**0.002**	0.098	0.050	0.051	**0.216**	**0.058**	**<0.001**
Gender, female	-0.154	0.244	0.529	-0.444	0.616	0.472	0.521	0.736	0.480
BMI, kg/m^2^	0.015	0.029	0.621	0.089	0.074	0.229	0.163	0.088	0.066
Medication, nr^a^	0.055	0.035	0.118	0.062	0.089	0.488	0.165	0.106	0.122
Karnofsky, score^b^	**-0.031**	**0.011**	**0.008**	**-0.066**	**0.030**	**0.026**	**-0.075**	**0.036**	**0.036**
Cancer, stage^c^	0.129	0.130	0.325	0.284	0.329	0.389	0.636	0.393	0.107

SM: skeletal muscle; IMAT: intermuscular adipose tissue; SD: standard deviation; *β*: beta; SE: standard error; kg: kilograms; m: meters; nr: number. Data available in a subgroup of ^a^ N = 252; ^b^ N = 238; ^c^ N = 245. When log-transformed values of percentage IMAT surface area were used to calculate intra-individual heterogeneity, p-values of age and Karnofsky score were not statistically significant. P-values of <0.05 were considered statistically significant and are indicated in bold.

### 3.4 Measures of specific muscle groups and intra-individual heterogeneity predicting adverse outcomes

As shown in Table 5, percentage of SM surface area was not associated with severe postoperative complications in any of the muscle groups. Higher muscle density was significantly associated with a lower risk of severe postoperative complications in both the lateral muscles and the psoas muscle, also after adjustment for possible confounders. Higher percentage IMAT surface area in the lateral muscles was significantly associated with a higher risk of severe postoperative complications in the crude and adjusted analysis. Higher intra-individual heterogeneity of percentage of SM surface area was associated with a higher risk of severe postoperative complications in the crude model, but not in the adjusted model.

**Table 5:** Association between measures of specific muscle groups and severe postoperative complications.

Muscle measures	Severe postoperative complications					
	Crude model			Adjusted model		
	OR	95% CI	p-value	OR	95% CI	p-value
*Rectus abdominis muscle*						
SM, %	0.881	0.733–1.058	0.174	0.931	0.768–1.129	0.466
Muscle density, HU	0.979	0.951–1.008	0.160	0.982	0.951–1.013	0.255
IMAT, %	1.016	0.995–1.038	0.139	1.012	0.989–1.035	0.302
*Lateral muscles*						
SM, %	1.083	0.980–1.198	0.119	1.115	1.000–1.244	0.050
Muscle density, HU	**0.952**	**0.911–0.995**	**0.028**	**0.952**	**0.908–0.998**	**0.039**
IMAT, %	**1.051**	**1.007–1.097**	**0.023**	**1.057**	**1.011–1.106**	**0.015**
*Psoas muscle*						
SM, %	0.924	0.793–1.077	0.314	0.920	0.782–1.083	0.316
Muscle density, HU	**0.946**	**0.906–0.987**	**0.011**	**0.944**	**0.901–0.989**	**0.014**
IMAT, %	1.113	0.894–1.387	0.339	1.133	0.895–1.434	0.298
*Back muscles*						
SM, %	0.996	0.909–1.092	0.930	0.964	0.876–1.061	0.456
Muscle density, HU	0.985	0.943–1.028	0.489	0.974	0.930–1.021	0.277
IMAT, %	1.009	0.963–1.056	0.720	1.020	0.970–1.073	0.445
*Intra-individual heterogeneity*						
SM %, SD	**1.246**	**1.019–1.523**	**0.032**	1.199	0.966–1.489	0.100
Muscle density, HU	1.002	0.923–1.086	0.969	0.985	0.903–1.076	0.741
IMAT %, SD	1.057	0.996–1.120	0.066	1.050	0.986–1.119	0.128

SM: skeletal muscle; HU: Hounsfield units; IMAT: intermuscular adipose tissue; SD: standard deviation; OR: odds ratio; CI: confidence interval. The right and left side of muscle groups were grouped in analyses. Severe postoperative complications was defined as 0) no or grade 1–2 Clavien-Dindo complications; 1) grade 3–5 Clavien-Dindo complications. Adjusted model (N = 245): adjusted for gender, age and stage of cancer. Similar results were found if log-transformed values of IMAT were used to calculate intra-individual heterogeneity of IMAT %. P-values of <0.05 were considered statistically significant and are indicated in bold.

The results of the cox regression analysis are given in Table 6. None of the measures of specific muscle groups were significantly associated with long-term survival.

**Table 6:** Association between measures of specific muscle groups and long-term survival.

Muscle measures	Long-term survival					
	Crude model			Adjusted model		
	HR	95% CI	p-value	HR	95% CI	p-value
*Rectus abdominis muscle*						
SM, %	0.978	0.891–1.075	0.649	1.008	0.918–1.107	0.863
Muscle density, HU	0.991	0.976–1.006	0.231	0.996	0.981–1.012	0.639
IMAT, %	0.999	0.986–1.013	0.928	0.996	0.983–1.009	0.561
*Lateral muscles*						
SM, %	1.019	0.964–1.077	0.512	1.005	0.948–1.066	0.859
Muscle density, HU	0.979	0.958–1.001	0.059	0.988	0.965–1.011	0.296
IMAT, %	1.016	0.992–1.041	0.203	1.009	0.982–1.035	0.526
*Psoas muscle*						
SM, %	0.953	0.877–1.036	0.255	0.955	0.878–1.039	0.283
Muscle density, HU	0.992	0.970–1.014	0.474	0.996	0.973–1.020	0.758
IMAT, %	0.969	0.837–1.121	0.670	0.975	0.833–1.140	0.747
*Back muscles*						
SM, %	1.009	0.960–1.060	0.728	1.010	0.961–1.062	0.687
Muscle density, HU	0.985	0.963–1.008	0.196	0.993	0.969–1.018	0.585
IMAT, %	1.016	0.993–1.039	0.183	1.004	0.978–1.031	0.767
*Intra-individual heterogeneity*						
SM %, SD	1.066	0.956–1.188	0.251	1.034	0.924–1.156	0.562
Muscle density, HU	1.011	0.969–1.055	0.613	1.000	0.957–1.045	0.990
IMAT %, SD	1.009	0.975–1.044	0.617	0.995	0.959–1.033	0.807

SM: skeletal muscle; HU: Hounsfield units; IMAT: intermuscular adipose tissue; SD: standard deviation; HR: hazard ratio; CI: confidence interval. The right and left side of muscle groups were grouped in analyses. The hazard ratio for death is given, survival time was calculated in months. Adjusted model (N = 245): adjusted for gender, age and stage of cancer. Similar results were found if log-transformed values of IMAT were used to calculate intra-individual heterogeneity of IMAT %.

## 4 Discussion

CT-based muscle measures differed between specific abdominal muscle groups and showed intra-individual heterogeneity in a cohort of older colon cancer patients, indicating that total cross-sectional measures are not representative of specific abdominal muscle groups. Although the rectus abdominis muscle had the lowest percentage of SM surface area and muscle density and the back muscles the highest percentage IMAT surface area, these were not associated with adverse outcomes, neither was intra-individual heterogeneity. Lower muscle density of the psoas muscle and lateral muscles, as well as higher percentage IMAT surface area of the lateral muscles, were associated with severe postoperative complications.

In our recent study on the association between CT-based muscle measures and the risk of adverse outcomes in older colorectal cancer patients, several associations between varying muscle measures and outcome measures were found, however, none of the total abdominal muscle measures were consistently or statistically significantly associated with surgery-related complications, dose-limiting toxicity or overall survival. The discrepancy with existing literature may be due to analysis of continuous muscle measures instead of using population-based cut-off points, proper inclusion of solely older patients and adjusting for multiple testing. Differences in the effects of age and inactivity on specific muscle groups have been found in previous studies [5, 6, 9]. Therefore, CT scan analysis of specific muscles to predict the risk of adverse outcomes in cancer patients might be worthwhile. Several studies have aimed to predict adverse outcomes and reduce CT scan analysis time by solely analyzing the psoas muscle [21, 22, 23] but the results were inconclusive. Psoas muscle area was found not to be representative of total SM surface area in ovarian [24] and colorectal cancer patients [22] and cannot be claimed to be representative of overall skeletal muscle mass [25]. Morphological explanations for the identification of a specific muscle group to predict adverse outcomes are lacking. The current study hypothesized the muscle with the highest percentage IMAT surface area and lowest muscle density to be most predictive of adverse outcomes, since muscle atrophy is thought to be replaced by intermuscular fat [26], and IMAT and muscle density are highly correlated [18].

Previous studies in individuals without cancer found that the psoas muscle showed the least amount of fat infiltration compared to other abdominal muscles [5, 27, 28], which was confirmed in our study. Deep, paraspinal and back muscles, including the transversus abdominis muscle, psoas muscle, erector spinae and quadratus lumborum muscles, stabilize the trunk and are continuously active in upright position [7]. While superficial muscles, including the rectus abdominis and external- and internal oblique muscles, are activated during particular movements of the trunk [7]. Therefore, superficial muscles were expected to be most affected by atrophy and intermuscular fat infiltration and have the lowest muscle density. This was also shown in studies on the effect of age on the morphology of specific muscle groups, which identified superficial muscles and mainly the rectus abdominis muscle to be affected by atrophy and intermuscular fat infiltration [5, 6, 8]. Muscle density was indeed lowest in the superficial rectus abdominis muscle, but on the contrary IMAT was highest in the back muscles. Patients may have become more inactive during the preceding year in which their cancer was diagnosed, resulting in increased IMAT in the back muscles including the erector spinae muscle [10]. This was supported by a relatively high percentage IMAT in the back muscles in our cohort compared to other cohorts [27, 28]. However, unexpectedly, neither muscle density of the rectus abdominis muscle nor percentage IMAT surface area of the back muscles, was associated with adverse outcomes in this cohort. This could be because measures of specific muscle groups do not take the intra-individual heterogeneity that exists between muscles into account [7, 29]. The current study confirmed the large intra-individual heterogeneity that exists within individuals in percentage of SM surface area, muscle density and percentage IMAT surface area. More specifically, intra-individual heterogeneity varied considerably between individuals as is shown by the large range of SDs that was found for each muscle measure and especially for percentage IMAT surface area. However, intra-individual heterogeneity was not associated with adverse outcomes after adjusting for possible confounders.

Both muscle density and percentage IMAT surface area of the lateral muscles were associated with severe postoperative complications, indicating the relevance of muscle quality measures. As the lateral muscles did not have the lowest muscle density of highest IMAT, this may be explained by the effects of age and inactivity that can occur over time. Oblique muscles were most affected with age and inactivity in a study on atrophy of trunk muscles comparing young individuals and older residents of nursing homes [9], although another study in community-based individuals of 40–90 years old identified the highest age-related effect on muscle density and muscle size in the rectus abdominis muscle [6]. Our results suggest a potential benefit of physical interventions aimed specifically at improving the muscle quality of lateral muscles to decrease the risk of postoperative complications in older cancer patients. Physical exercise intervention programs have been proved effective in reducing IMAT in specific muscle groups [30]. As longitudinally measuring total abdominal muscle measures using CT scan analysis in colorectal cancer patients has shown promising results [4, 31], future research should focus on longitudinally measuring specific muscle groups, to assess whether a higher decline in SM and muscle density or increase in IMAT in specific muscle groups is the main risk factor of adverse outcomes in cancer patients. Moreover, it would be interesting to combine CT-measured muscle parameters with nutritional markers as well as physical performance tests. Both sarcopenia [13, 32], diagnosed by a combination of low skeletal muscle mass and low physical function, and malnutrition [33], are two prevalent geriatric syndromes in older cancer patients that could be of high importance in identifying older cancer patients with a high risk of adverse outcomes. Another interesting factor would be the association between systemic inflammatory markers and individual muscle groups as it has been suggested that systemic inflammation may also be associated with skeletal muscle measures [34] and the risk of adverse outcomes [35, 36].

This study addressed measures of specific muscle groups with high accuracy. Systematic errors may have occurred in the identification of muscle boundaries. The lateral muscles consisting of external- and internal oblique and transversus abdominis muscles and the back muscles including the erector spinae and quadratus lumborum muscles, could not be identified separately. Taking these muscles together could have evened out differences that can be expected between superficial and deep abdominal muscles. Occasionally, CT scan analysis was difficult if the psoas muscle was not directly situated on top of the processus transversus, which could have led to an overestimation of IMAT surface area. Considering the surface area of IMAT was notably low within the psoas muscle it is considered unlikely that this affected the results. Due to the number of patients with severe postoperative complications (N = 29) overfitting of the adjusted model may be present as three clinically relevant confounders of gender, age and stage of cancer were considered in the adjusted analysis. These analyses warrant further verification by larger cohorts.

## 5 Conclusions

CT-based muscle measures differed between specific abdominal muscle groups and showed large intra-individual heterogeneity in a cohort of older colon cancer patients. These results indicate that total cross-sectional abdominal muscle measures do not adequately represent the heterogeneity that exists between specific abdominal muscle groups within an individual. Lower muscle density and higher percentage IMAT surface area of the lateral muscles were associated with severe postoperative complications. There may be added value in analyzing the decline in muscle density and IMAT surface area by CT scan analysis of specific muscle groups to predict the risk of adverse outcomes in older cancer patients, however, this needs further underpinning by prospective, longitudinal studies.

## Declarations

### Author contribution statement

S. Looijaard: Conceived and designed the experiments; Performed the experiments; Analyzed and interpreted the data; Wrote the paper.

A. Maier, J. Klaase and C. Meskers: Conceived and designed the experiments; Analyzed and interpreted the data; Contributed reagents, materials, analysis tools or data; Wrote the paper.

D. Bouman: Conceived and designed the experiments; Contributed reagents, materials, analysis tools or data; Wrote the paper.

A. Voskuilen and T. van Zanten: Performed the experiments; Analyzed and interpreted the data; Wrote the paper.

### Funding statement

This work was supported by European Union's Horizon 2020 research and innovation program under the Marie- Sklodowska- Curie (grant agreement no. 675003) (PANINI program) and by an unrestricted grant of the 10.13039/501100001782University of Melbourne, Australia, received by Professor Andrea B. Maier.

### Declaration of interests statement

The authors declare no conflict of interest.

### Additional information

No additional information is available for this paper.
